# Extending telestroke to broader teleneurology in sparsely populated regions: real-world experience from a German network

**DOI:** 10.3389/fstro.2026.1981787

**Published:** 2026-10-27

**Authors:** Maike Kern, Tim Bastian Braemswig, Stephan Kinze, Malgorzata Kotlarz-Böttcher, Heinrich J. Audebert, Janina R. Behrens

**Affiliations:** 1 Department of Neurology, Charité – Universitätsmedizin Berlin, Corporate Member of Freie Universität Berlin and Humboldt-Universität zu Berlin, Berlin, Germany; 2 Center for Stroke Research Berlin, Charité – Universitätsmedizin Berlin, Corporate Member of Freie Universität Berlin and Humboldt-Universität zu Berlin, Berlin, Germany; 3 Klinik für Neurologie, BG Klinik Unfallkrankenhaus Berlin, Berlin, Germany; 4 Department of Neurology, University Hospital Greifswald, Greifswald, Germany

**Keywords:** ANNOTeM, hub-and- spoke network, rural healthcare, teleneurology, telestroke

## Abstract

**Introduction:**

Telestroke networks provide established infrastructure for specialist neurological care in rural regions, but their real-world use beyond stroke remains insufficiently characterized. We aimed to describe the diagnostic spectrum of acute teleconsultations within a comprehensive teleneurology network in sparsely populated and structurally underserved regions of Northeast Germany and to explore factors associated with the distribution of diagnoses.

**Methods:**

We conducted a retrospective observational analysis of acute teleneurological consultations performed within the ANNOTeM network at 12 continuously participating hospitals between January 2023 and December 2024. Consultations were classified into seven predefined diagnostic categories. Diagnostic distribution was analyzed descriptively; univariable binary and exploratory multinomial logistic regression models were used to examine associations with patient-, hospital-, and regional-level characteristics.

**Results:**

Of 4,697 consultations, 4,207 had a single diagnostic category. Suspected stroke accounted for 60.0%, whereas 40.0% concerned non-stroke presentations, reaching 48.3% at individual hospitals (range 30.8–48.3%). More than 70% of non-stroke cases received another neurological diagnosis rather than a non-neurological diagnosis. Median patient age was 70 years. Increasing age was significantly associated with neurological diagnosis (OR per 10 years 1.09, 95% CI 1.04–1.15; *p* < 0.001) and suspected stroke (OR 1.42, 95% CI 1.38–1.48; *p* < 0.001). A higher regional proportion of residents aged 60–80 years was significantly associated with neurological diagnosis (OR 1.04, 95% CI 1.00–1.07; *p* = 0.039). A higher density of neurology-related outpatient specialists was significantly associated with a lower relative likelihood of another neurological diagnosis compared with suspected stroke (OR 0.94, 95% CI 0.89–0.98; *p* = 0.008).

**Discussion:**

Overall, these findings demonstrate that our network, built on an established telestroke infrastructure, is frequently used for comprehensive acute teleneurology in routine clinical care. The substantial non-stroke workload, consisting mostly of neurological diagnoses, together with its associations with demographic structure and specialist availability, highlights the growing relevance of comprehensive teleneurology in aging and underserved regions. Existing telestroke networks may therefore provide a practical platform for bridging geographical and temporal gaps in neurological specialist care beyond stroke.

## 1 Introduction

Limited access to neurological expertise remains a major challenge for healthcare systems worldwide (Dorsey et al., 2018; Behrens J. et al., 2026; Almallouhi et al., 2020). Even in high-income countries, specialist neurological services are often concentrated in urban centers, leaving rural and structurally underserved regions with limited access (Dorsey et al., 2018; Behrens J. et al., 2026; Audebert et al., 2005; Barlinn et al., 2021; Ickenstein et al., 2010; Masouris et al., 2024; Behrens J. R. et al., 2026). This is particularly relevant for acute neurological conditions such as stroke, status epilepticus, or acute spinal cord injury, in which rapid diagnosis and treatment strongly influence patients' long-term outcomes (Behrens J. et al., 2026; Barlinn et al., 2021; Ickenstein et al., 2010; Masouris et al., 2024; Behrens J. R. et al., 2026; Amadi-Obi et al., 2014; Hubert et al., 2019; Audebert et al., 2006; Erdur et al., 2023; Müller-Barna et al., 2014).

Telestroke has become an established model for providing neurological expertise over geographical distances and is now an integral component of organized stroke care in many rural regions (Dorsey et al., 2018; Almallouhi et al., 2020; Barlinn et al., 2021; Ickenstein et al., 2010; Behrens J. R. et al., 2026; Amadi-Obi et al., 2014; Hubert et al., 2019; Erdur et al., 2023). Telemedical stroke networks have enabled safe thrombolysis in hospitals without on-site neurological expertise and have been associated with improved clinical outcomes in rural settings (Behrens J. et al., 2026; Audebert et al., 2005; Barlinn et al., 2021; Ickenstein et al., 2010; Masouris et al., 2024; Behrens J. R. et al., 2026; Hubert et al., 2019; Audebert et al., 2006; Erdur et al., 2023; Müller-Barna et al., 2014). In Germany, nationwide hub-and-spoke telestroke infrastructure has developed from early initiatives such as the Telemedical Project for Integrative Stroke Care in Southeast Bavaria (TEMPiS) (Behrens J. et al., 2026; Audebert et al., 2005; Barlinn et al., 2021; Masouris et al., 2024; Behrens J. R. et al., 2026; Audebert et al., 2006; Müller-Barna et al., 2014). By 2021, 22 German networks connected 43 coordinating centers with 225 cooperating hospitals, serving approximately 48 million people, with more than 38,000 teleconsultations performed in 2018 and utilization continuing to increase thereafter (Barlinn et al., 2021; Masouris et al., 2024). Within this established infrastructure, more than one in ten patients with stroke in Germany receive telemedical care (Barlinn et al., 2021). Although stroke remains the predominant indication, acute teleneurology also encompasses epilepsy, vertigo, headache, movement disorders, stroke mimics, and other neurological emergencies (Dorsey et al., 2018; Behrens J. et al., 2026; Almallouhi et al., 2020; Barlinn et al., 2021; Erdur et al., 2023; Wiborg and Widder, 2003). Evidence for these broader applications is growing, although it remains less robust than for telestroke (Dorsey et al., 2018; Behrens J. et al., 2026; Almallouhi et al., 2020; Behrens J. R. et al., 2026; Erdur et al., 2023).

Extending this established model, the Acute Neurological Care in Northeast Germany with Telemedicine Support (ANNOTeM) network provides telemedical specialist care for a broader spectrum of acute neurological emergencies beyond stroke (Behrens J. et al., 2026; Hubert et al., 2019; Erdur et al., 2023). This comprehensive model is particularly relevant in the regions served by ANNOTeM. Mecklenburg-Western Pomerania, Brandenburg, and Saxony-Anhalt are predominantly rural and differ markedly from major urban areas in both demographic structure and healthcare provision (Table 1). Population density is very low, whereas the proportion of older residents and the old-age dependency ratio are substantially higher than in Berlin, Frankfurt am Main, and western German non-city states (Hubert et al., 2019; Statistisches Bundesamt, 2025a,b,c). At the same time, outpatient physician density is approximately half that of the urban comparator regions, neurological specialist density is markedly lower, and a considerably larger proportion of neurology-related outpatient specialists are older than 65 years (Table 1) (KBV, 2026). Modeled travel-time data further indicate that large parts of Northeast Germany are located more than 30 min by road from a certified stroke unit or telestroke network hospital, highlighting persistent geographical barriers to timely specialist care (Barlinn et al., 2021).

**Table 1:** Demographic characteristics and outpatient physician density in the districts served by hospitals participating in the ANNOTeM network, with Berlin and Frankfurt am Main included as urban reference regions (Statistisches Bundesamt, 2025a,b,c; KBV, 2026).

					All physicians		Primary care physicians		Neurology-related specialists	
Partner hospital	Federal state	Hospital district	Population density, inhabitants/km^2^	Population aged 60–80 years, %	Physician density, per 100,000 inhabitants	Physicians aged >65 years, %	Physician density, per 100,000 inhabitants	Physicians aged >65 years, %	Physician density, per 100,000 inhabitants	Physicians aged >65 years, %
1	Saxony-Anhalt	Altmark district of Salzwedel	35	29.6	134.6	9.0	53.4	6.8	4.9	25.0
2	Saxony-Anhalt	Altmark district of Salzwedel	35	29.6	134.6	9.0	53.4	6.8	4.9	25.0
3	Saxony-Anhalt	Stendal	44	30.2	156.0	9.3	68.9	7.9	10.9	16.7
4	Saxony-Anhalt	Wittenberg	63	30.5	159.8	9.1	63.0	12.8	7.3	22.2
5	Brandenburg	Havelland	99	25.4	144.1	12.3	65.6	11.7	4.7	25.0
6	Brandenburg	Havelland	99	25.4	144.1	12.3	65.6	11.7	4.7	25.0
7	Brandenburg	Ostprignitz-Ruppin	39	29.8	164.2	14.0	73.1	16.4	7.0	14.3
8	Brandenburg	Teltow-Fläming	84	24.6	143.8	11.0	59.4	9.5	5.7	0.0
9	Brandenburg	Potsdam-Mittelmark	85	26.5	138.4	7.1	67.4	10.0	5.4	16.7
10	Brandenburg	Märkisch-Oderland	92	28.1	143.3	12.2	59.4	16.8	7.0	7.1
11	Brandenburg	Uckermark	38	31.2	168.0	12.1	63.6	10.7	8.5	20.0
12	Mecklenburg-Western Pomerania	Vorpommern-Rügen	67	30.6	168.7	9.4	72.9	11.4	6.6	6.7
**Median**			**65**	**29.6**	**144.1**	**10.2**	**64.6**	**11.05**	**6.15**	**18.35**
**Range**			**35–99**	**24.6–31.2**	**134.6–168.7**	**7.1–14**	**53.4–73.1**	**6.8–16.8**	**4.7–10.9**	**0–25**
**Berlin**			**3045**	**16.9**	**262.2**	**14.5**	**63.6**	**14.4**	**9.1**	**10.0**
**Frankfurt am Main**			**4136**	**19.2**	**248.2**	**16.4**	**70.7**	**17.2**	**10.8**	**12.6**

Population density is expressed as inhabitants per km^2^; physician and neurologist density are expressed as the number of physicians or neurologists per 100,000 inhabitants. Population and physician data are reported at district level. “Neurology-related specialists” corresponds to the German statutory health insurance physician group “Nervenärzte,” which includes specialists in neurology, psychiatry, and combined neurology and psychiatry. Berlin and Frankfurt am Main are shown for descriptive comparison and are not participating hospital locations.

These structural conditions create a particular need for neurological expertise beyond stroke to other acute neurological presentations in an aging population with limited local specialist capacity (Dorsey et al., 2018). Against this background, the present study aimed to characterize the real-world use of teleneurology within the ANNOTeM network by describing the diagnostic spectrum of acute teleconsultations and exploring associations with patient age, regional demographic structure, and local neurological service availability.

## 2 Materials and methods

### 2.1 Study design and setting

We conducted a retrospective observational analysis of acute teleneurological consultations within the Acute Neurological Care in Northeast Germany with Telemedicine Support network (ANNOTeM). ANNOTeM is organized as a hub-and-spoke network connecting three academic neurological centers with rural acute-care hospitals in Saxony-Anhalt, Brandenburg, and Mecklenburg-Western Pomerania (Behrens J. et al., 2026; Behrens J. R. et al., 2026; Erdur et al., 2023). Participating hospitals have 24/7 access to remote board-certified neurological expertise, video-based patient examination, real-time review of neuroimaging and clinical information, and immediate recommendations regarding local treatment or transfer for thrombectomy, neurosurgery, or other specialized care (Behrens J. et al., 2026; Hubert et al., 2019; Erdur et al., 2023). Quality management and assurance are embedded in routine network operations through regular interdisciplinary case and process reviews, standardized and continuously updated treatment pathways, continuous technical quality assurance, and twice-yearly structured quality-review meetings with each participating hospital.

The analysis included all 12 hospitals that participated continuously in the network between January 1, 2023, and December 31, 2024. All acute teleconsultations involving direct patient assessment during this period were eligible. Follow-up or repeat consultations relating to the same clinical episode, telephone-only case discussions without direct patient contact, and records lacking the minimum information required for inclusion were excluded. As the study analyzed routinely collected hospital data whose use for research purposes is permitted under the Berlin Hospital Act (“Berliner Krankenhausgesetz”), individual informed consent was not required.

### 2.2 Consultation characteristics

Clinical and administrative data were extracted retrospectively from routinely documented consultation records provided by the network technology provider (Meytec^®^, Werneuchen, Germany). Patient-level variables included age, sex, premorbid functional status, living situation before the index event, consultation time, use of video communication, time of symptom onset including onset of in-hospital disease manifestations, hospital admission time, and consultation start and end times. The time of hospital admission and the documented start and end of the consultation episode were used to calculate the recorded consultation duration.

### 2.3 Diagnostic classification

Based on the working diagnosis documented by the consulting neurologist, consultations were classified into seven predefined categories: (1) suspected stroke, (2) epileptic seizure or epilepsy-associated presentation, (3) traumatic brain injury, (4) acute spinal cord syndrome, (5) neuroinfectious disease, including meningitis and encephalitis, (6) other neurological diagnoses [peripheral vestibular disorders (e.g., Benign paroxysmal positional vertigo, vestibular neuritis), headache, Parkinson's disease/movement disorders, brain tumor, multiple sclerosis, peripheral nerve lesions, peripheral facial palsy, functional neurological disorders, and dementia/neurodegenerative disease], and (7) non-neurological diagnoses or stroke mimics [e.g., encephalopathy of other cause (hypertension, non-neurological infection, renal failure, dehydration), delirium, [pre-]syncope, electrolyte disturbance (e.g., hyponatremia), angina pectoris, psychiatric disorders, hypoglycemia, medication intoxication, deterioration of general condition].

More than one category could be assigned when the documented assessment included overlapping or competing diagnoses. Consultations with exactly one diagnostic category constituted the primary analysis population for analyses based on mutually exclusive diagnostic classification. Consultations with multiple categories were analyzed separately and described according to the recorded diagnostic combinations. Acute spinal cord syndromes and neuroinfectious diseases occurred only in combination with other diagnoses and were therefore not included as separate categories in the single-diagnosis analysis.

Consultations with exactly one diagnostic category constituted the primary analysis population for mutually exclusive diagnostic classification. Two binary diagnostic groupings were defined for subsequent regression analyses: suspected stroke vs. non-stroke presentations and neurological vs. non-neurological diagnosis. The neurological diagnosis outcome comprised suspected stroke (1) epileptic presentations, (2) traumatic brain injury, (3) and other neurological diagnoses, (6) whereas non-neurological diagnoses and stroke mimics (7) constituted the reference group.

### 2.4 Hospital and regional characteristics

Hospital-level variables included hospital capacity, annual consultation volume, and the availability of local neurological services. Local neurological care was categorized into three predefined levels: hospitals without on-site neurological services, internal medicine hospitals with neurological consultation or neurological presence during defined core hours, and hospitals with an independent neurological department.

Regional variables included the distance from each partner hospital to the nearest hospital with an independent neurological department, the number of neurological hospitals within a 50-km radius of each partner hospital, population density (inhabitants per km^2^), the regional proportion of residents aged 60–80 years (%), and the density of outpatient neurologists (number per 100,000 inhabitants) (Table 1). Outpatient neurologist density was based on the German statutory health insurance physician group “Nervenärzte,” comprising specialists in neurology, psychiatry, and combined neurology and psychiatry. Regional healthcare and population data were obtained from the National Association of Statutory Health Insurance Physicians, the Federal Clinic Atlas, and the Federal Statistical Office (Statistisches Bundesamt, 2025a,b,c; KBV, 2026).

### 2.5 Analytical strategy and statistical analysis

First, consultation data were analyzed descriptively to characterize the spectrum and frequency of teleneurological care beyond suspected stroke and its variation across participating hospitals. Categorical variables are presented as absolute and relative frequencies. Continuous variables were assessed descriptively and are reported as means with standard deviations (SD) or medians with interquartile ranges and ranges, as appropriate.

Second, univariable binary logistic regression analyses were performed for the two binary diagnostic outcomes defined above, neurological vs. non-neurological diagnosis and suspected stroke vs. non-stroke presentations. For the neurological diagnosis outcome, patient age, the regional proportion of residents aged 60–80 years (in percent), neurological service level of the network hospital, distance to the nearest neurological department (in km), density of outpatient neurology-related specialists (per 100,000 inhabitants), and consultation initiation time were each entered separately as predictors. Consultation initiation time was examined to explore whether teleconsultation use varied according to temporal availability of local neurological expertise. For the suspected stroke outcome, separate univariable models were calculated for patient age, the regional proportion of residents aged 60–80 years (in percent), distance to the nearest neurological department (in km), and density of outpatient neurology-related specialists (per 100,000 inhabitants). These analyses were intended to explore whether the likelihood of a neurological diagnosis or suspected stroke varied according to individual and regional age structure and neurological care availability. For analyses involving distance to the nearest neurological department, the hospital with its own neurological department was excluded. Patient age was entered per 10-year increase and distance to the nearest neurological department per 10-km increase to improve clinical interpretation.

Third, exploratory multinomial logistic regression analyses were additionally performed to further differentiate the associations examined with the binary diagnostic outcomes above. For patient age, the analysis was used to characterize how the relative likelihood of individual neurological diagnostic categories varied with increasing age, using non-neurological diagnoses or stroke mimics as the reference category for the density of outpatient neurology-related specialists, suspected stroke served as the reference category to explore whether regional differences in outpatient neurological care were associated with the relative frequency of specific non-stroke neurological presentations among teleconsultations, potentially reflecting greater emergency-care use where outpatient neurological capacity is limited. This comparison was chosen because outpatient neurological care is expected to be more directly related to the management of non-stroke neurological conditions, whereas stroke prevention and care are shared across several medical specialties.

Results are reported as odds ratios with 95% confidence intervals (CI). Model fit was assessed using Nagelkerke's pseudo-R^2^. All statistical tests were two-sided, and a *p*-value below 0.05 was considered statistically significant. Statistical analyses were performed using IBM SPSS Statistics, version 31.0.0.0 (IBM Corp., Armonk, NY, USA).

## 3 Results

### 3.1 Hospital and regional characteristics

The study included 12 hospitals participating in the ANNOTeM network across Saxony-Anhalt, Brandenburg, and Mecklenburg-Western Pomerania. Hospital size, consultation volume, local neurological services, and regional access to neurological care varied considerably between partner hospitals (Table 2). The median hospital capacity was 201.5 beds (range 90–357), and the median number of teleconsultations conducted in 2023 and 2024 was 309 per hospital (range 126–852).

**Table 2:** Hospital and regional characteristics of hospitals participating in the ANNOTeM network and teleneurological consultations conducted in 2023 and 2024.

Partner hospital	Beds	Neurological service level	Consultations	Number of Hospitals with neurological departement within 50 km	Distance to nearest hospital with neurological departement, km
1	180	IM	520	2	43.95
2	207	IM	596	1	33.50
3	357	Neurology Dept.	264	5	33.08
4	300	IM + NC	126	3	29.13
5	145	IM + NC	369	5	28.22
6	235	IM + NC	656	25	38.13
7	169	IM	212	3	37.60
8	259	IM + NC	271	13	40.06
9	255	IM	324	26	28.67
10	167	IM	213	25	34.48
11	90	IM	294	5	36.30
12	196	IM	852	3	32.47
**Median (Range)**	**201.5 (90–357)**		**309 (126–852)**	**5** **(1–26)**	**33.99 (28.22–43.95)**

Data are shown for each participating hospital. Neurological service level: IM, internal medicine hospital without on-site neurological services; IM + NC, internal medicine hospital with neurological consultation or neurological presence during defined core hours. Neurology Dept.: hospital with own neurological department. Three participating hospitals are located in peri-urban areas directly adjacent to a major urban region, resulting in a comparatively high number of neurological hospitals within a 50-km radius. Nevertheless, these hospitals also serve patients from sparsely populated and structurally underserved areas extending away from the urban center. The final row presents medians and ranges for continuous variables.

Most participating departments were internal medicine departments without on-site neurology services, whereas only one hospital had an independent neurology department. Regional access to neurological care was heterogeneous, with on average (median) five hospitals with neurology department within a 50-km radius (range 1–26). The mean distance to the nearest such hospital was 34.6 km (SD 4.8; range 28.2–44.0 km). Detailed hospital level and regional characteristics are presented in Table 2.

### 3.2 Study population and consultation characteristics

A total of 5,680 teleneurological consultations were initially recorded. Following application of the predefined exclusion criteria, 4,697 consultations from 12 hospitals were included in the final study population. Of these, 4,634 consultations were available for diagnosis-based analyses, including 4,207 assigned to a single predefined diagnostic category and 427 assigned to multiple categories; 63 consultations had no documented predefined diagnostic category (Table 3). Video consultation was performed in 95.0% of cases (Table 3).

**Table 3:** Study population and clinical characteristics of teleneurological consultations conducted within the ANNOTeM network in 2023 and 2024.

Characteristic	Category	Value	Range
Study population, *n*	Consultations initially recorded	5,680	
	Consultations included in the study population	4,697	
	Consultations without a documented predefined diagnostic category	63 (1.3%)	
Diagnosis-based analyses, *n*	Consultations with one predefined diagnostic category	4,207 (89.6%)	
	Consultations with more than one predefined diagnostic category	427 (9.1%)	
	Consultations included in diagnosis-based analyses	4,634 (98.7%)	
Consultation modality, *n*	Video consultation	4,400 (95.0%)	
Consultation duration, minutes, median	Overall	103 [IQR 76]	5–1552
Sex, *n*	Female	2,379 (51.3%)	
	Male	2,255 (48.7%)	
Age, years, median	Overall	70 [IQR 24]	16–102
Age by single diagnosis, years, median	Suspected stroke	73 [IQR 63–82]	20–99
	Epileptic presentation	63 [IQR 44–77]	18–96
	Traumatic brain injury	79 [IQR 56–84]	18–95
	Other neurological diagnosis	59 [IQR 40–72]	16–102
	Non-neurological diagnosis/mimic	67 [IQR 52–80]	16–99
Premorbid functional status, *n*	mRS score 0	1,835 (65.4%)	
Previous living situation, *n*	Independent at home	2,848 (61.5%)	
	Receiving care at home	360 (7.8%)	
	Care institution	283 (6.1%)	
	Unknown	1,143 (24.7%)	
Symptom onset, *n*	In-hospital onset	829 (17.9%)	

Data are presented as *n* (%), median [interquartile range], or range, as appropriate. Percentages for sex refer to consultations included in the diagnosis-based analyses (*n* = 4,634); percentages for premorbid mRS refer to patients with available mRS data (*n* = 2,806). IQR, interquartile range; mRS, modified Rankin Scale.

Among the 4,634 consultations included in the diagnosis-based analyses, sex distribution was balanced, and median patient age was 70 years (IQR 24; range 16–102) (Table 3). Age distributions varied across diagnostic categories, with suspected stroke and traumatic brain injury occurring in older patients than epileptic, other neurological, and non-neurological presentations (Figure 1, Table 3). Premorbid functional status and living situation are summarized in Table 3.

**Figure 1:**
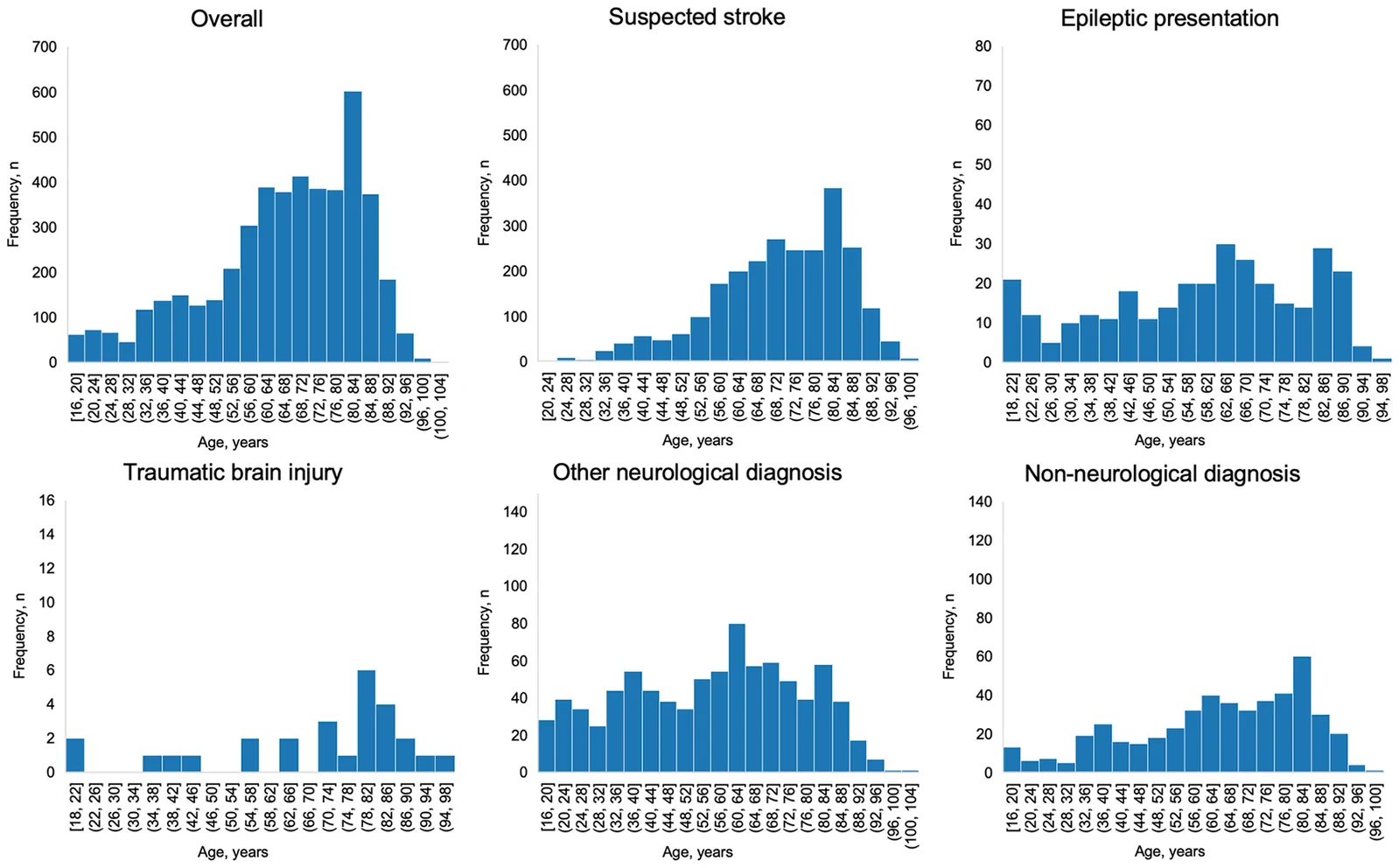
Age distributions among all teleconsultations assigned to a single diagnostic category. Histograms were reconstructed from the data. Patient age is grouped into age ranges and displayed on the x-axis. The traumatic brain injury distribution should be interpreted cautiously because of the small number of cases.

Consultations showed two circadian peaks, with 1,014 consultations (21.9%) initiated between 11 AM and 1:59 PM and 963 (20.8%) between 16 PM and 6:59 PM (Figure 2). After stratification by local neurological services, however, each group showed a single predominant peak: hospitals without onsite neurological services peaked between 11:00 and 13:59 (762 consultations, 25.6%), whereas hospitals with local neurological services peaked between 16:00 and 18:59, accounting for 24.9% of consultations (413 consultations) (Figure 2).

**Figure 2:**
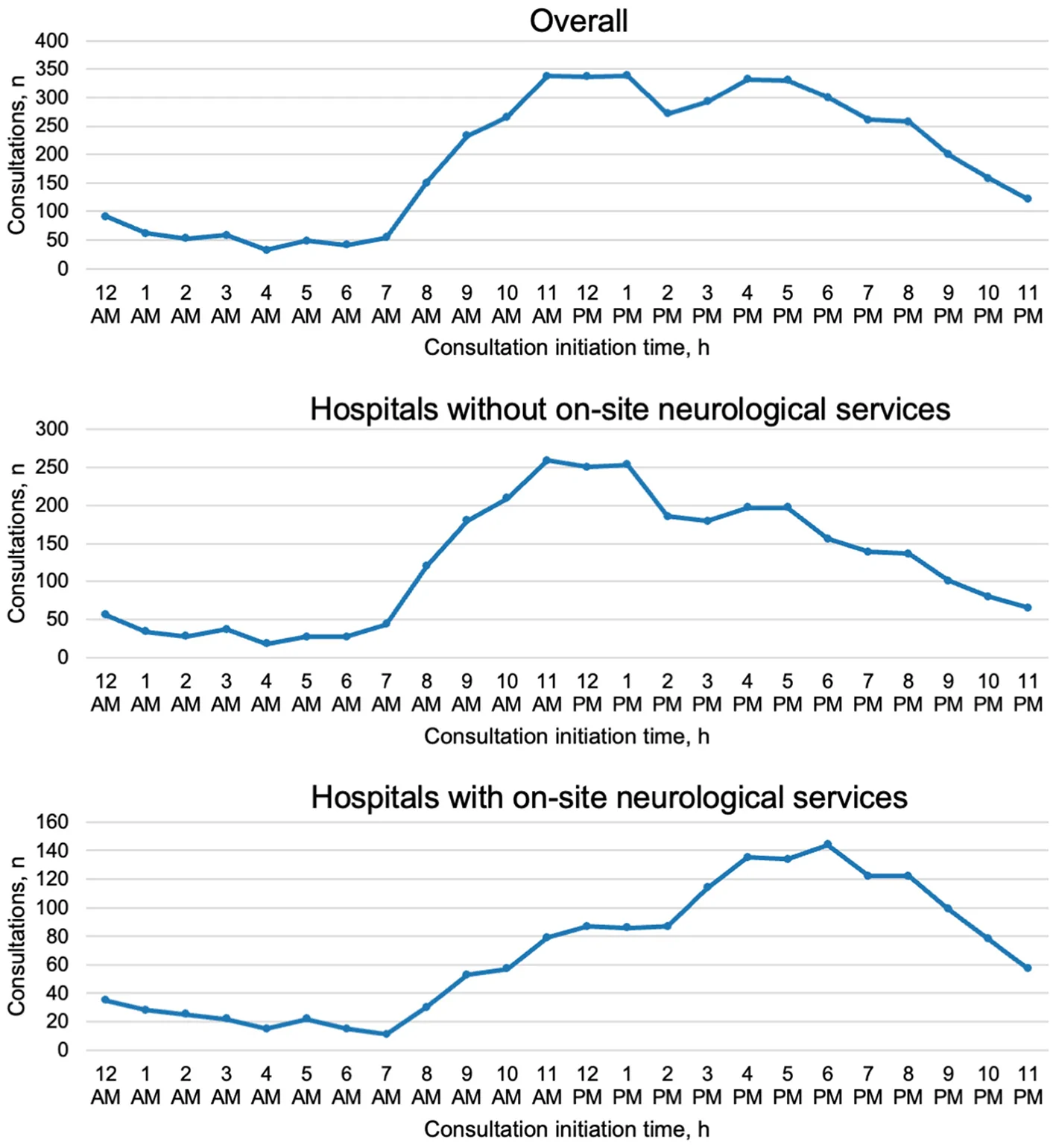
Hourly distribution of consultation initiation overall (*n* = 4634) and stratified by the availability of local neurological services. Hospitals with local neurological services included hospitals with an independent neurological department or neurological consultation/presence during defined core hours (*n* = 1657). Hospitals without any on-site neurological service had *n* = 2977.

The median consultation duration was 103 min (IQR 76, range 5–1552), reflecting a markedly right-skewed distribution due to individual extreme values (Table 3). In-hospital symptom onset was documented in 829 cases (17.9%) (Table 3).

### 3.3 Distribution of diagnoses

Among consultations assigned to a single principal diagnostic category, suspected stroke was most frequent, accounting for 2,523 cases (60.0%) (Table 4). Other neurological diagnoses represented 20.3% of consultations, followed by non-neurological diagnoses or stroke mimics (11.6%), epileptic presentations (7.5%), and traumatic brain injury (0.6%) (Table 4). The distribution varied across participating centers, with center-specific proportions ranging from 51.7 to 69.2% for suspected stroke and from 15.7 to 25.9% for other neurological diagnoses (Table 4). Accordingly, diagnoses other than suspected stroke accounted for up to 48.3% of single-diagnosis consultations at individual centers (Table 4).

**Table 4:** Distribution of single diagnostic categories across hospitals participating in the ANNOTeM network in 2023 and 2024.

Partner hospital	Suspected stroke	Epileptic presentation	Traumatic brain injury	Other neurological diagnosis	Non-neurological diagnosis/stroke mimic	Total
1	273 (57.7%)	43 (9.1%)	3 (0.6%)	101 (21.4%)	53 (11.2%)	473
2	302 (57.5%)	46 (8.8%)	4 (0.8%)	111 (21.1%)	62 (11.8%)	525
3	161 (66.5%)	16 (6.6%)	0 (0.0%)	39 (16.1%)	26 (10.7%)	242
4	69 (59.0%)	14 (12.0%)	2 (1.7%)	16 (13.7%)	16 (13.7%)	117
5	177 (54.0%)	21 (6.4%)	3 (0.9%)	76 (23.2%)	51 (15.5%)	328
6	313 (54.2%)	48 (8.3%)	10 (1.7%)	134 (23.2%)	72 (12.5%)	577
7	104 (55.0%)	10 (5.3%)	0 (0.0%)	49 (25.9%)	26 (13.8%)	189
8	145 (60.2%)	14 (5.8%)	2 (0.8%)	47 (19.5%)	33 (13.7%)	241
9	187 (64.0%)	11 (3.8%)	0 (0.0%)	59 (20.2%)	35 (12.0%)	292
10	126 (64.9%)	7 (3.6%)	1 (0.5%)	42 (21.6%)	18 (9.3%)	194
11	137 (51.7%)	26 (9.8%)	2 (0.8%)	60 (22.6%)	40 (15.1%)	265
12	529 (69.2%)	61 (8.0%)	0 (0.0%)	120 (15.7%)	54 (7.1%)	764
**Overall**	**2,523 (60.0%)**	**317 (7.5%)**	**27 (0.6%)**	**854 (20.3%)**	**486 (11.6%)**	**4,207**
Range	51.7–69.2%	3.8–12.0%	0–1.7%	15.7–25.9%	7.1–15.5%	—

Data are presented as *n* (row %). Percentages refer to consultations assigned to exactly one predefined diagnostic category within each hospital. The range refers to the minimum and maximum row percentages across the 12 hospitals.

Among the 427 consultations assigned to multiple diagnostic categories, the most frequent combinations were suspected stroke with another neurological diagnosis (36.8%), another neurological diagnosis with a non-neurological diagnosis or mimic (19.9%), and suspected stroke with a non-neurological diagnosis or mimic (16.9%) (Figure 3). The remaining diagnostic combinations were less common and are shown in Figure 3.

**Figure 3:**
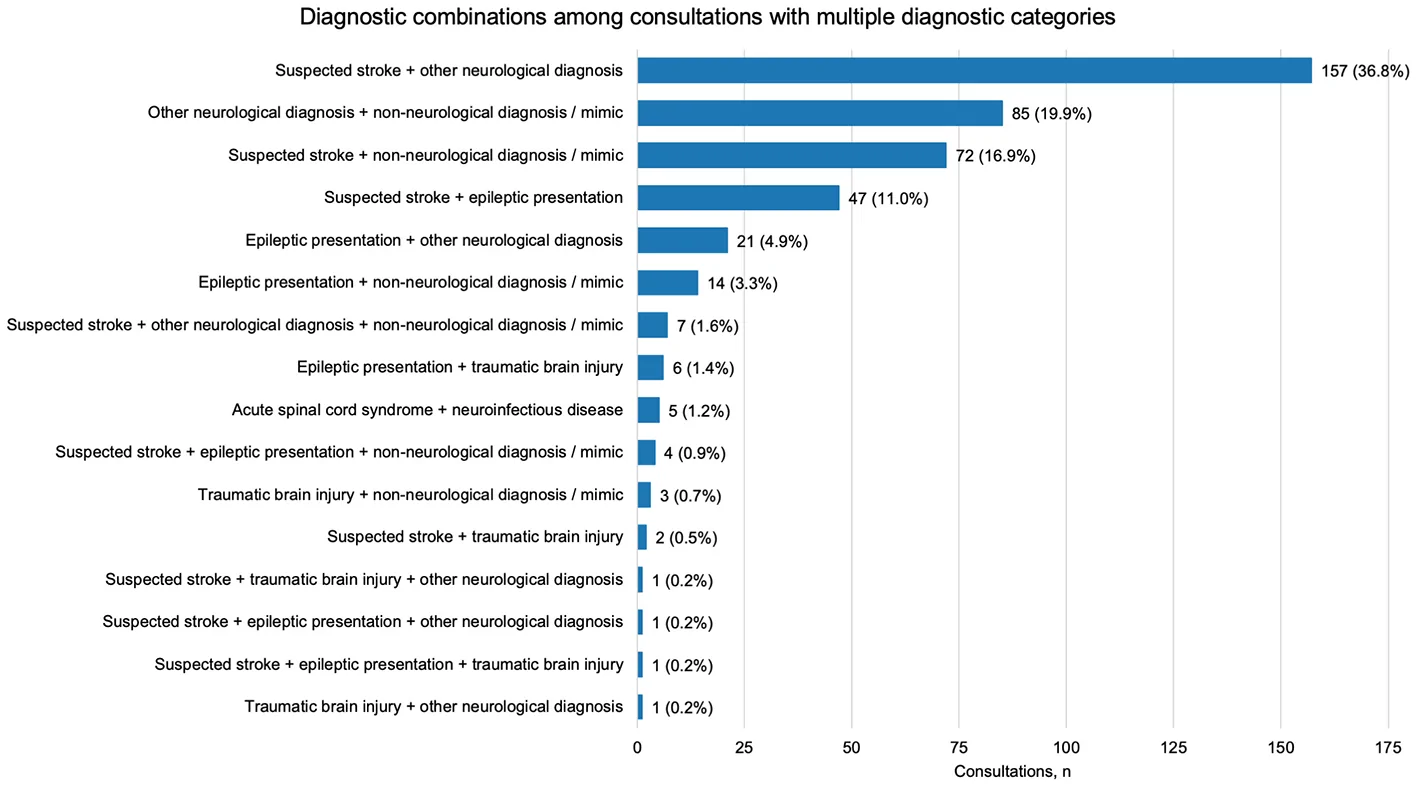
Distribution of diagnostic combinations among consultations assigned to multiple diagnostic categories (*n* = 427).

### 3.4 Factors associated with a neurological diagnosis

Figure 4 summarizes the univariable associations with a neurological diagnosis. Increasing patient age (OR per 10 years 1.09, 95% CI 1.04–1.15; *p* < 0.001) and a higher regional proportion of residents aged 60–80 years (OR per percentage point 1.04, 95% CI 1.00–1.07; *p* = 0.039) were associated with higher odds of a neurological diagnosis. Local neurological service level was also associated with the presence of a neurological diagnosis (overall *p* = 0.035). Neurological diagnoses were identified more often in hospitals with neurological consultation or core-hour expertise than in hospitals without local neurological expertise (OR 0.77, 95% CI 0.63–0.94; *p* = 0.010), while no significant difference was observed compared with hospitals with a Department of Neurology (OR 0.94, 95% CI 0.62–1.44; *p* = 0.779).

**Figure 4:**
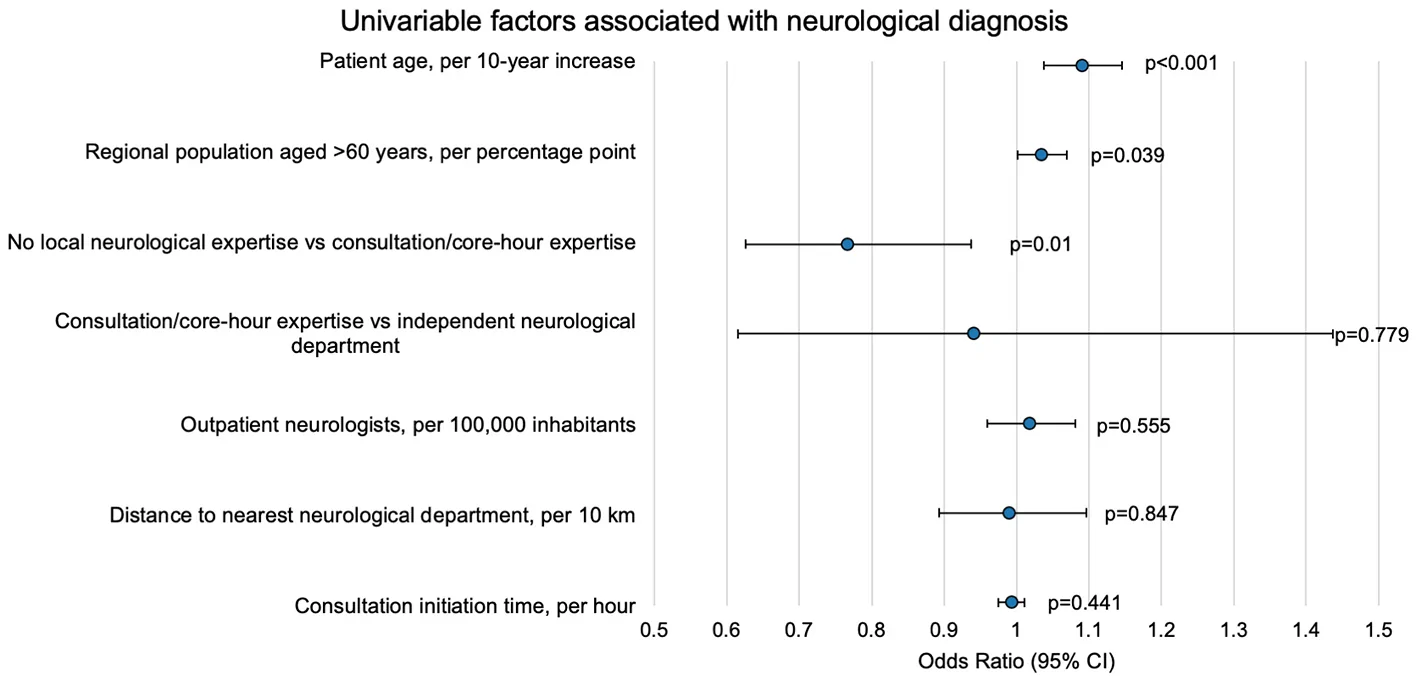
Forest plot of univariable factors associated with neurological diagnosis. Odds ratios (ORs), 95% confidence intervals (CIs) and p-values are shown. Consultation initiation time: OR per hour 0.99 (95% CI 0.98–1.01; Nagelkerke's R^2^ < 0.001). Distance to the nearest neurological department: OR per 10 km 0.99 (95% CI 0.89–1.10; R^2^ < 0.001). Outpatient neurologist density: OR 1.02 (95% CI 0.96–1.08; R^2^ < 0.001). Consultation/core-hour expertise versus an independent neurological department: OR 0.94 (95% CI 0.62–1.44; R^2^ = 0.003). No local neurological expertise versus consultation/core-hour expertise: OR 0.77 (95% CI 0.63–0.94; R^2^ = 0.003). Regional population aged >60 years: OR per percentage point 1.04 (95% CI 1.00–1.07; R^2^ = 0.002). Patient age: OR per 10 years 1.09 (95% CI 1.04–1.15; R^2^ = 0.005).

The likelihood of diseases being identified as neurological diagnosis and daytime of consultation initiation were not associated with distance to a hospital with a neurological department, the density of neurology-related outpatient specialists, and were not associated with whether a neurological diagnosis was identified. The explanatory power of all univariable models was low, with Nagelkerke's R^2^ values ranging from < 0.001 to 0.005 (Figure 4).

### 3.5 Factors associated with suspected stroke

Figure 5 summarizes the univariable associations with suspected stroke. Patient age showed the strongest association, with 42.4% higher odds per 10-year increase (OR 1.42, 95% CI 1.38–1.48; *p* < 0.001). Greater distance from the partner hospital to the nearest hospital with a Department of Neurology was associated with slightly lower odds of suspected stroke (OR per 10 km 0.93, 95% CI 0.87–0.99; *p* = 0.023), whereas a higher regional proportion of residents aged 60–80 years was associated with slightly higher odds (OR per percentage point 1.02, 95% CI 1.00–1.05; *p* = 0.028). The density of outpatient neurologists was not significantly associated with suspected stroke (OR 0.97, 95% CI 0.91–1.04; *p* = 0.435). Explanatory power was low for the regional structural factors (Nagelkerke's R^2^ = 0.001–0.002) and higher, while the age model showed a higher but still limited value (R^2^ = 0.121; Figure 5).

**Figure 5:**
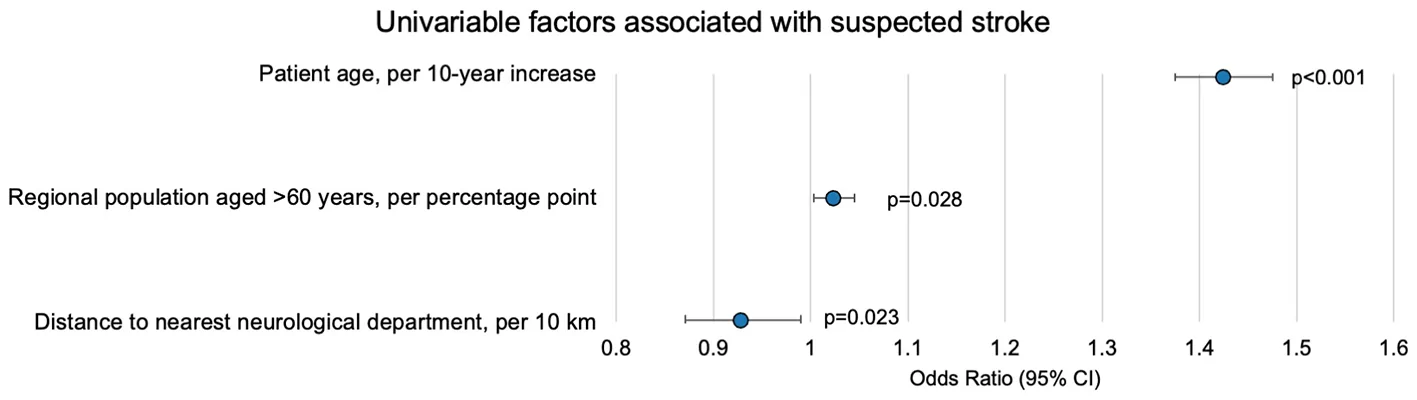
Forest plot of univariable factors associated with suspected stroke. Odds ratios (ORs), 95% confidence intervals (CIs) and p-values are shown. Distance to the nearest neurological department: OR per 10 km 0.93 (95% CI 0.87–0.99; Nagelkerke's R^2^ = 0.002). Regional population aged >60 years: OR per percentage point 1.02 (95% CI 1.00–1.05; R^2^ = 0.001). Patient age: OR per 10 years 1.42 (95% CI 1.38–1.48; R^2^ = 0.121).

### 3.6 Multinomial analysis of diagnostic group

Figure 6 shows the association between patient age and diagnostic group, using non-neurological diagnoses or mimics as the reference category. Per 10-year increase in age, the relative likelihood of suspected stroke increased by 32%, while epileptic presentations and other neurological diagnoses showed 10% and 17% lower relative odds, respectively. The explanatory value of the model was moderate (Nagelkerke's pseudo-R^2^ = 0.132).

**Figure 6:**
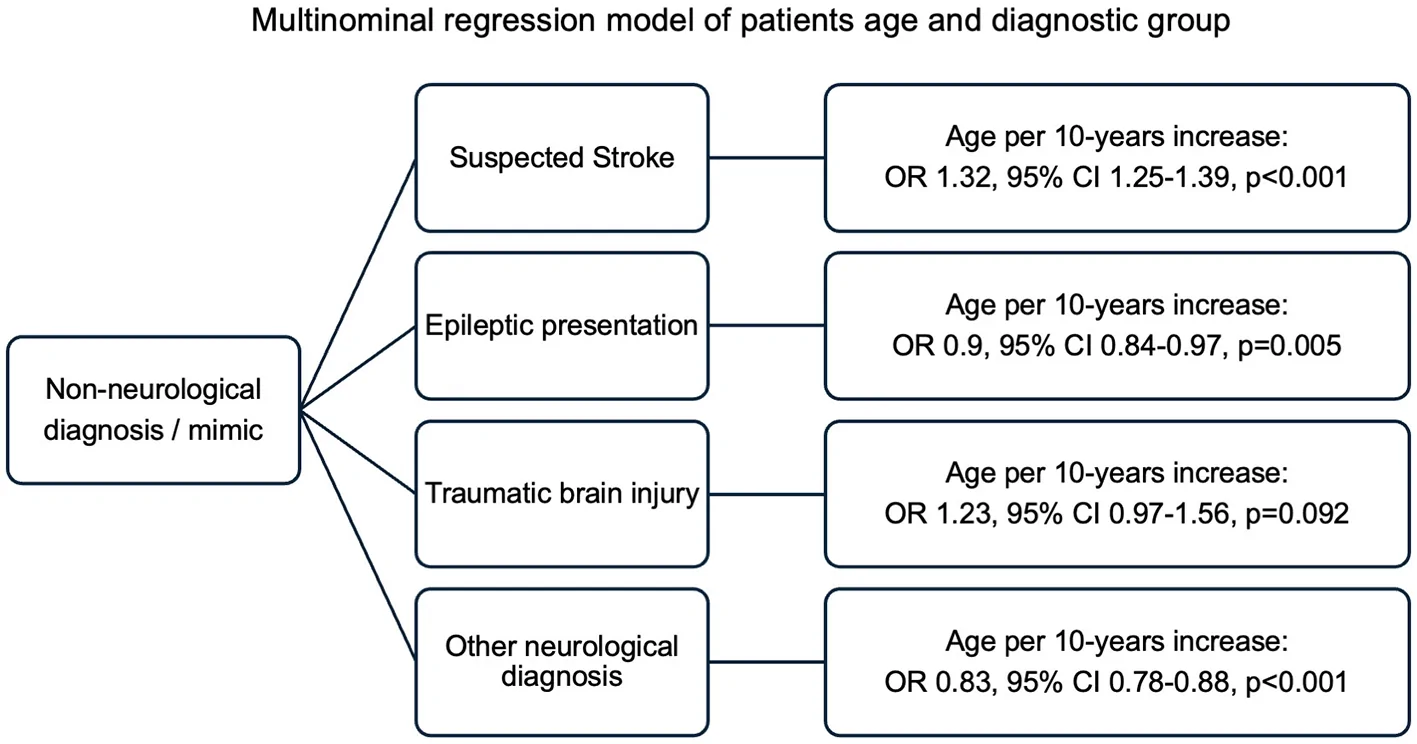
Association of patient age with diagnostic group in the multinomial regression model (reference category: non-neurological diagnosis / stroke mimic). Estimates are presented as Odds Ratio (OR) per 10-year increase in age with 95% confidence intervals (CIs) and *p*-values. The overall explanatory value of the model was Nagelkerke's pseudo-R^2^ = 0.132.

Figure 7 summarizes the association between the density of neurology-related outpatient specialists and diagnostic group, using suspected stroke as the reference category. A higher density of neurology-related outpatient specialists was significantly associated with a 6% lower relative likelihood of another neurological diagnosis compared with suspected stroke. No significant associations were observed for epileptic presentations or non-neurological diagnoses and mimics. The explanatory value of the model was very low (Nagelkerke's pseudo-R^2^ = 0.004).

**Figure 7:**
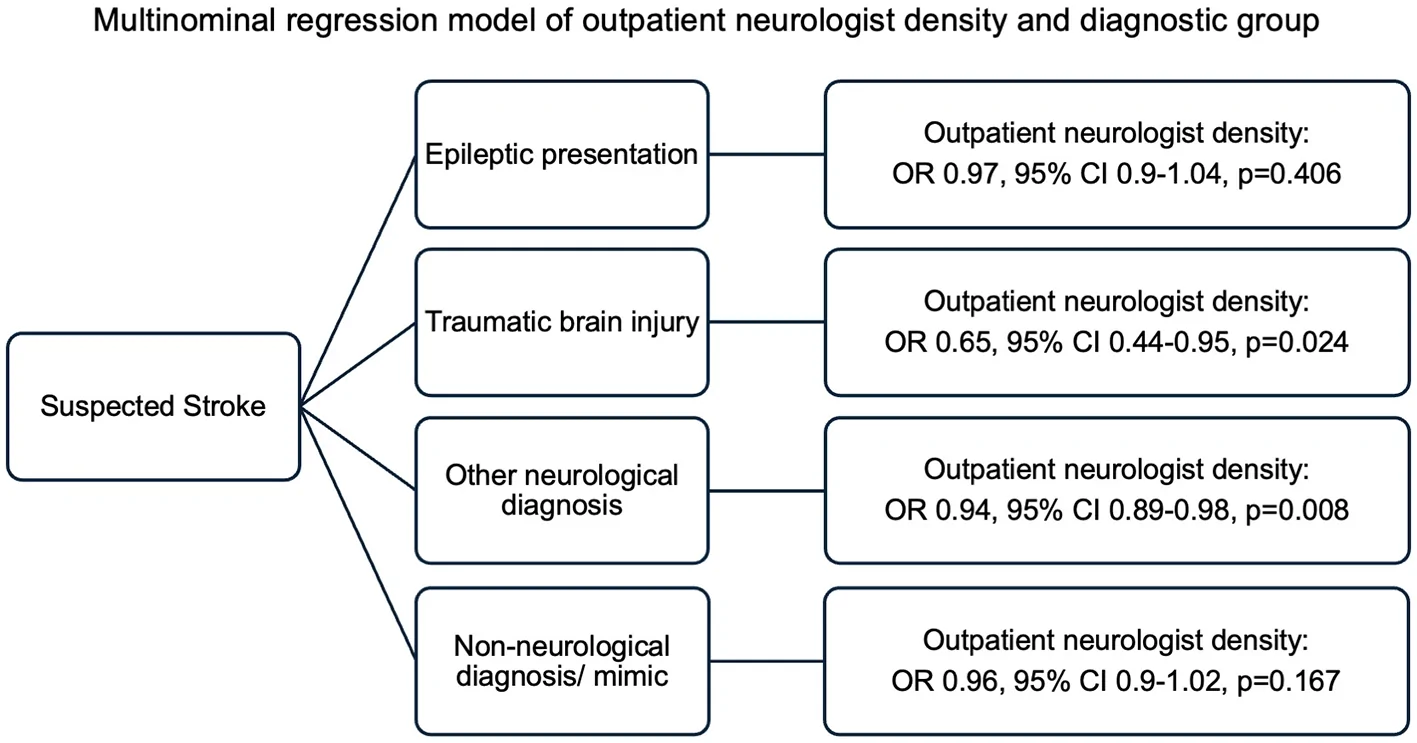
Association of outpatient neurologist density with diagnostic group in the multinomial regression model (reference category: suspected stroke). Odds ratios (OR) are shown per additional outpatient neurology-related specialist per 100,000 inhabitants with 95% confidence intervals (CIs) and *p*-values. The overall explanatory value of the model was Nagelkerke's pseudo-R^2^ = 0.004.

The findings for traumatic brain injury should be interpreted cautiously because of the small number of cases in this diagnostic category.

## 4 Discussion

In this two-year study of acute teleneurological care in rural and structurally underserved regions, the diagnostic case mix demonstrates that a telemedicine infrastructure originating from the telestroke model is routinely used for a substantially broader spectrum of acute care needs beyond stroke: four in ten single-category consultations involved non-stroke presentations, with proportions reaching 48% at individual hospitals. About 70% of the non-stroke consultations involved another neurological diagnosis or epileptic presentation, rather than non-neurological diagnoses or stroke mimics. This case mix was observed across all 12 participating hospitals and occurred in an older population with a median age of 70 years, in whom increasing age was associated with a greater likelihood of neurological diagnosis and suspected stroke. Consultation patterns also differed according to local neurological service availability, while exploratory analyses suggested associations with regional population structure and outpatient neurological capacity.

Previous studies have already suggested substantial demand for specialist assessment beyond stroke in hospitals without on-site neurology (Almallouhi et al., 2020; Ickenstein et al., 2010). In a rural US teleneurology network, cerebrovascular disease accounted for approximately one-third of consultations, while seizures and other neurological disorders constituted much of the remaining workload (Almallouhi et al., 2020). German telestroke experience has likewise shown that a meaningful proportion of patients referred for suspected stroke ultimately have alternative diagnoses or stroke mimics (Barlinn et al., 2021). The present study extends these observations by examining a network deliberately designed to provide comprehensive acute teleneurology rather than stroke assessment alone. Our data show that non-stroke consultations are not an occasional secondary use of the service but represent a consistent component of routine network activity.

Telestroke networks already provide most of the structures required for broader acute teleneurology, including continuous specialist availability, secure audiovisual communication, rapid access to neuroimaging, standardized treatment pathways, and established transfer arrangements. Extending these infrastructures to additional neurological emergencies may therefore represent a pragmatic approach to specialist care. This may be particularly relevant in sparsely populated regions, where maintaining sufficient case volumes for disease-specific services can be challenging and catchment areas may need to cover large geographical distances (Hubert et al., 2019; Müller-Barna et al., 2014; Wiborg and Widder, 2003). In addition to bridging geographical distance, the consultation patterns in our study suggest that teleneurology also complements local neurological services across temporal gaps in specialist availability. Hospitals without on-site neurological expertise showed their highest consultation volume between 11 AM and 1:59 PM, whereas hospitals with local neurological services showed a distinct peak between 4 PM and 6:59 PM. As several network hospitals also provide consultation-based neurological coverage during core working hours on weekends, further differentiation between weekdays and weekends was not considered informative. This temporal pattern may indicate greater need of teleneurology after locally available consultation or core hours end in hospitals with partial neurological coverage. Moreover, hospitals with consultation-based or core-hour neurological services did not differ significantly from hospitals with an independent neurological department in the likelihood of a neurological diagnosis, whereas hospitals without any local neurological expertise showed significantly lower odds. This association may reflect differences in the threshold for suspecting a neurological condition, referral patterns, patient populations, or other unmeasured hospital-level factors. Although these findings derive from univariable analyses and should not be interpreted causally, they suggest that existing telestroke infrastructure can support comprehensive teleneurology by complementing local specialist care both geographically and temporally.

The importance of this expanded role may increase further when demographic change and outpatient specialist capacity are considered. The median age of the study population was 70 years, and increasing age was associated with a greater likelihood of a neurological diagnosis, with an even stronger association observed for suspected stroke. At the regional level, each additional percentage point in the proportion of residents aged 60–80 years was associated with approximately 4% higher odds of a neurological diagnosis. This is particularly relevant because almost 30% of residents in the regions served by ANNOTeM are aged 60–80 years, nearly twice the proportion in the urban comparator regions (Table 1). At the same time, 40% of single-category consultations involved diagnoses other than suspected stroke, indicating that neurological care demand extends well beyond cerebrovascular disease. This broad case mix occurs in regions with comparatively limited specialist capacity: the density of neurology-related outpatient specialists is approximately one-third lower than in the urban comparator regions, and a substantially higher proportion of these specialists are older than 65 years (Table 1). The association between a higher density of neurology-related outpatient specialists and a lower relative likelihood of another neurological diagnosis compared with suspected stroke had very low explanatory value and may reflect differences in outpatient access, referral pathways, or other unmeasured regional factors. One possible explanation is that better outpatient neurological access may reduce emergency presentations of non-stroke neurological conditions through earlier recognition and management, whereas stroke prevention and occurrence are less directly linked to outpatient neurological care. These cross-sectional data do not allow conclusions about whether changes in outpatient specialist capacity would alter future consultation demand. Nevertheless, the combination of an older regional population, a broad neurological case mix, and limited specialist availability supports the relevance of comprehensive teleneurology in rural regions.

Most other regional associations were small and explained little of the observed variation in diagnostic classification. Greater distance to the nearest hospital with an independent neurological department was associated with slightly lower odds of suspected stroke. Given the narrow distance range and very low explanatory value of the model, this counterintuitive finding should be interpreted cautiously and may reflect differences in emergency medical service routing, preselection, or other unmeasured regional factors rather than an effect of distance itself.

Previous evaluation of the ANNOTeM network showed that implementation of a managed teleneurological care system was associated with improved outcomes across a broad spectrum of neurological emergencies (Behrens J. et al., 2026; Erdur et al., 2023). The current study addresses a complementary question by describing which patients and diagnoses generate teleconsultations in routine care. With 40% of single-category consultations concerning conditions other than suspected stroke, a comprehensive service must maintain expertise and pathways for diagnostic uncertainty, seizure-related presentations, other acute neurological conditions, and decisions regarding local treatment or transfer. Recorded consultation intervals were highly variable and included implausible extreme values; they should therefore not be interpreted as direct measures of active consultation time or resource use. Consultation numbers alone may not fully capture the resources required, while quality indicators and reimbursement frameworks centered predominantly on reperfusion treatment and stroke-unit processes may not adequately capture this broader workload. Staffing, financing, and quality standards should consequently reflect the actual spectrum and complexity of neurological presentations managed within these networks (Erdur et al., 2023).

The observed case mix therefore provides an empirical basis for future evaluations of comprehensive teleneurology as a regional care strategy. Such studies should examine patient-centered outcomes, treatment decisions, avoidable transfers, health-economic effects, and whether broader use of existing telestroke infrastructure for teleneurology improves the sustainability of specialist services in low-density regions.

The strengths of this study include its multicenter design, 2-year observation period, large number of consultations, and inclusion of hospitals with different levels of local neurological support. Several limitations should be considered. First, the retrospective analysis depended on the completeness and accuracy of routine documentation. The recorded categories represented consultation-time working diagnoses rather than independently adjudicated final discharge diagnoses. Second, “other neurological diagnoses” formed a broad and clinically heterogeneous category, limiting conclusions about individual conditions. In addition, traumatic brain injury was represented by only a small number of cases and acute spinal cord syndromes as well as neuroinfectious diseases occurred in only five patients and exclusively in combination with other diagnostic categories, precluding meaningful separate analyses of these groups. Third, 427 consultations had more than one diagnostic category and could not be assigned unambiguously to the mutually exclusive stroke vs. non-stroke comparison; the principal 60% vs. 40% estimate was therefore restricted to the 4,207 single-category consultations. Fourth, some patient-level clinical and functional information was unavailable, and recorded consultation intervals included implausible extreme values and may not represent active consultation time. Fifth, regression analyses were exploratory and univariable. Their explanatory value was generally low and reached moderate levels only in selected age-related models. Consultations were also clustered within 12 hospitals, while hospital- and region-level characteristics were shared by multiple consultations; standard errors for these exploratory associations may therefore be underestimated. No adjustment for multiple testing was applied. Associations with regional characteristics should therefore not be interpreted causally and may reflect unmeasured differences in referral pathways, patient populations, or local healthcare structures. All analyses were restricted to patients who received a teleconsultation and therefore cannot be generalized to the overall patient population treated at the participating hospitals. Finally, this study did not include treatment, transfer, outcome, or cost data. Although previous ANNOTeM evaluations found improved 90-day outcomes after network implementation without a relevant increase in 90-day costs compared with non-network hospitals (Behrens J. et al., 2026; Erdur et al., 2023), the present analysis cannot determine whether comprehensive teleneurology beyond stroke contributed to these effects. Generalizability may also be limited to networks serving comparable rural and structurally underserved settings in Germany.

## 5 Conclusion

In 12 hospitals serving sparsely populated and neurologically underserved regions, four in ten single-category acute teleconsultations concerned non-stroke presentations, predominantly neurological conditions rather than stroke mimics. This broad case mix, together with associations with patient age and regional age structure, highlights the relevance of broadening the application of telestroke to comprehensive acute teleneurology in aging and rural populations. Established telestroke infrastructure may therefore provide a pragmatic platform for acute neurological care beyond stroke.

## Data Availability

The data analyzed in this study is subject to the following licenses/restrictions: The dataset cannot be made publicly available because it contains sensitive patient-level clinical data and is subject to applicable data protection and confidentiality regulations. Requests to access these datasets should be directed to maike.kern@charite.de.
